# Current data processing methods and reporting standards for untargeted analysis of volatile organic compounds using direct mass spectrometry: a systematic review

**DOI:** 10.1007/s11306-024-02104-3

**Published:** 2024-03-16

**Authors:** K Rosenthal, MR Lindley, MA Turner, E Ratcliffe, E Hunsicker

**Affiliations:** 1 School of Sport, Exercise & Health Sciences, Loughborough University, Loughborough, UK; 2 School of Health Sciences, Faculty of Medicine and Health, University of New South Wales, Sydney, Australia; 3 Department of Chemistry, Loughborough University, Loughborough, UK; 4 Department of Chemical Engineering, Loughborough University, Loughborough, UK; 5 Department of Mathematical Sciences, Loughborough University, Loughborough, UK

**Keywords:** Direct mass spectrometry, Data processing, Untargeted metabolomics, Reporting standards, Bioinformatics

## Abstract

**Introduction:**

Untargeted direct mass spectrometric analysis of volatile organic compounds has many potential applications across fields such as healthcare and food safety. However, robust data processing protocols must be employed to ensure that research is replicable and practical applications can be realised. User-friendly data processing and statistical tools are becoming increasingly available; however, the use of these tools have neither been analysed, nor are they necessarily suited for every data type.

**Objectives:**

This review aims to analyse data processing and analytic workflows currently in use and examine whether methodological reporting is sufficient to enable replication.

**Methods:**

Studies identified from Web of Science and Scopus databases were systematically examined against the inclusion criteria. The experimental, data processing, and data analysis workflows were reviewed for the relevant studies.

**Results:**

From 459 studies identified from the databases, a total of 110 met the inclusion criteria. Very few papers provided enough detail to allow all aspects of the methodology to be replicated accurately, with only three meeting previous guidelines for reporting experimental methods. A wide range of data processing methods were used, with only eight papers (7.3%) employing a largely similar workflow where direct comparability was achievable.

**Conclusions:**

Standardised workflows and reporting systems need to be developed to ensure research in this area is replicable, comparable, and held to a high standard. Thus, allowing the wide-ranging potential applications to be realised.

## Introduction

A high-throughput non-invasive metabolomics approach can have many applications across clinical, industrial, and academic fields including disease identification (Pereira et al., 2022; Wehinger et al., 2007), food spoilage (Fang et al., 2022; Franke & Beauchamp, 2017), and plant chemistry (Xiao et al., 2022). Analysis of gas-phase samples such as breath or headspace allows for a non-invasive non-destructive protocol. Direct mass spectrometry (MS) methods can analyse volatile organic compounds (VOCs) in gas-phase samples within seconds or continuously monitor a sample to identify changes over time, often without any need for pre-treatment steps (Perez-Hurtado et al., 2017; Rosenthal et al., 2019, 2021; Trefz et al., 2013). Alongside the appropriate data processing and statistical methods, direct MS can provide an almost instantaneous metabolic profile which can be used to differentiate between sample types (Bregy et al., 2018; Hicks et al., 2015). This paper will provide an overview of current data processing and statistical methods used for direct MS and propose future research to improve the repeatability and impact of research aimed at gas-phase sample classification.

Comprehensive metabolomic approaches typically include chromatographic separation prior to mass analysis which requires more time and laboratory space. Gas Chromatography-Mass Spectrometry (GC-MS), which is widely considered the gold-standard for analysing gas-phase VOCs (Alkhalifah et al., 2020), has long run times (> 1 h). This means continuous monitoring is not possible using GC-MS and sample throughput is severely limited. Additionally, GC-MS requires complex preparation steps which may be inhibitive for non-expert use (Tait et al., 2014). The main advantage of employing chromatographic separation alongside MS is that there are two axes on which to distinguish compounds, and therefore GC-MS allows for accurate identification of analytes. However, the identity of compounds may not be necessary for many practical applications which may only require a yes or no answer, such as disease diagnostics.

Tandem-MS (commonly referred to as MS/MS or MS^n^) is a multiple stage analysis where smaller fragments are analysed at each subsequent stage and is often used to obtain structural information about specific molecules (Li et al., 2021). While tandem- and GC-MS are useful for researchers to identify compounds and explore metabolic pathways, a practical diagnostic application can be obtained using a metabolic profile consisting of *m/z* values and intensity counts where the true identity and concentration is unknown (Martinez-Lozano Sinues et al., 2015; Rosenthal et al., 2021). This profile, with appropriate data processing and statistical analysis, can then be used to accurately differentiate between sample types. Direct MS methods can obtain a metabolic profile in seconds, using cheaper instrumentation compared to tandem- or GC-MS, without any expert knowledge required to prepare samples and operate instrumentation.

Time-of-flight (TOF) or ion trap mass analysers can have a mass resolution high enough to enable identification through accurate mass-to-charge ratio (*m/z*) measurement, while maintaining a high throughput if used with direct infusion (Weber et al., 2020; Zielinski et al., 2018). However, these instruments tend to be large and expensive, which may be inhibitive for many practical applications, such as clinical diagnostics. Quadrupole mass analysers are typically smaller and cheaper; however, generally quadrupole mass analysers do not have a high enough resolution to distinguish between compounds with a similar *m/z*, with many only accurate to unit mass (1 Da), although the instruments may provide measurements to multiple decimal places (Ouyang & Cooks, 2009). For this reason, researchers often bin measurements into windows of 1 *m/z* width (Bunge et al., 2008; Fedrigo et al., 2010; Shestivska et al., 2011), which should increase the likelihood that measurements of the same compound are grouped together and reduce processing time and memory required to analyse the data (Finch et al., 2022).

For certain MS methods, such as proton transfer reaction (PTR) and selected ion flow tube (SIFT) ionisation, known reaction rates can be used to calculate the concentration of a compound within a sample (Lechner et al., 2005; Španěl & Smith, 1996); however, the compounds must first be identified, which may not be possible for untargeted analysis. When the actual concentrations are unknown, the results can be analysed qualitatively—whether or not a m/z window records an intensity above baseline—or ‘semi-quantitively’, such as using the relative intensities (fold change) to statistically differentiate between sample types. For semi-quantitative analysis, the reliability of intensity counts remains important to accurately differentiate between samples. This requires robust data processing workflows to ensure that the metabolic profiles are quantitatively reliable at the fold-change or other relative level.

Many published data processing workflows for MS are focussed on chromatography-coupled methods, such as GC-MS or liquid chromatography (LC)-MS (Di Guida et al., 2016; Dunn et al., 2011; Mak et al., 2014), as are many of the existing software tools designed for metabolomics analysis (Spicer et al., 2017). Kirwan et al. (2014) published a benchmark for processing direct infusion MS data in 2014 for liquid phase samples. The method includes probabilistic quotient normalisation, a technique designed to correct for dilution effects, which may not be relevant to gas-phase samples. Additionally, representative quality control samples were created by pooling the all the collected samples, which may not be practical or possible for gas-phase samples such as breath, which is often analysed by the participant breathing directly into the instrument.

Recent developments have been made in creating data processing tools specifically for PTR-TOF-MS, with the open-source PTRwid released in 2015 (Holzinger, 2015), the commercial Ionicon Data Analyzer released in 2020, and the open-source ptairMS released in 2021 (Roquencourt et al., 2022). Additionally, Cappellin et al. published a recommended data processing workflow for PTR-TOF-MS in 2011, with the MATLAB functions available on request. This paper has been cited 161 times (according to Google Scholar, accessed 14/06/2023) which suggests some uptake within the research community; however, without reading every paper, the use of the workflow and whether it has been applied to different ionisation and mass analyser methodologies cannot be confirmed. Workflows must be used consistently to ensure replicable and comparable research is produced.

Some workflows may have been designed for a particular type of direct MS but may still be relevant to other methods. Soft ionisation methods, such as PTR and SIFT-MS, should create similar datasets, as they typically produce ions with a mass-to-charge ratio of one higher or one lower than the mass of the original compound. However, some methods may only be relevant to a subset of ionisation methods or mass analysers; for example, TOF analysers have a higher mass resolution and therefore processes to obtain an exact mass for identification can be performed.

Metabolomics is a fast-growing field and MS methods can produce large amounts of data. User-friendly data processing and statistical tools are becoming increasingly available, allowing researchers to perform complex analyses without requiring expertise. However, these tools are not necessarily suited for every data type, and it is not yet generally clear which data processing methods give the best results in which situations. Biological data is intrinsically complex and metabolomics research in particular is difficult to reproduce among different labs without strict controls and standardised methods (Cambiaghi et al., 2017). Although a few studies have compared and developed metabolomic data processing workflows, the broader applicability and use within research has not been confirmed. This paper provides a systematic review of current use of data processing and analysis workflows of direct MS research as a critical first step to the development of description of best practice workflows for direct MS analysis.

## Objectives

1. Conduct a systematic review that summarises and characterises the data processing and analytic workflows currently in use for untargeted soft ionisation direct MS metabolomic research and examine whether there are common data processing methods for certain instrumentation and sample types.

2) Examine whether methodological reporting in the literature is sufficient to allow for replication of workflows.

3) Propose further work to ensure untargeted soft ionisation direct MS metabolomic research is replicable and comparable.

## Methods

### Reporting protocol

This systematic review was conducted using the principles of the Preferred Reporting Items for a Systematic Reviews and Meta-Analyses (PRISMA) (Page et al., 2021).

### Inclusion criteria

Articles were included in the study if they:

- Employed a direct mass spectrometry method.
- Performed untargeted metabolomics.
- Used a soft ionisation method.
- Analysed gas-phase volatile organic compounds.

### Exclusion criteria

Articles were excluded from the study if they:

- Did not present original mass spectrometry data.
- Performed only tandem mass spectrometry.
- Only used mass spectrometry in combination with a separation method such as chromatography.
- Were not available in English.

### Search methods

Searches were performed in Web of Science and Scopus databases. The search was limited to papers published after 2014 and up to and including 25th July 2022. The terms “mass spec”, “metabo”, and volat” were required to appear in either the title, abstract or keywords. Papers were excluded if the terms “chromatograph”, “tandem mass spec”, “GC-MS”, “LC-MS”, or “review” appeared in the title, abstract or keywords. The citations identified were imported into Covidence. Duplicates were identified using the Covidence online software and removed. The abstracts were then screened by searching for the terms ‘GC’ and ‘LC’, and each appearance manually checked to ensure it referred to chromatography before exclusion.

The remaining articles were then read in full and included if they met the inclusion/exclusion criteria. Duplicates not identified by the software were removed manually. The criteria were designed to ensure the practical methods were similar enough as to not require significantly differing data processing methods. Each article was screened by one author.

### Data extraction

The data was extracted by a single author. Information regarding publication (author and publication year), sample type, data collection methods, data processing methods, statistical analysis, software and packages used to process the data, cited data processing workflows, and the data and code availability. A separate quality assessment was not performed, as the overall objectives include areas typically covered by a quality assessment.

## Results and discussion

### Study selection

**Fig. 1:**
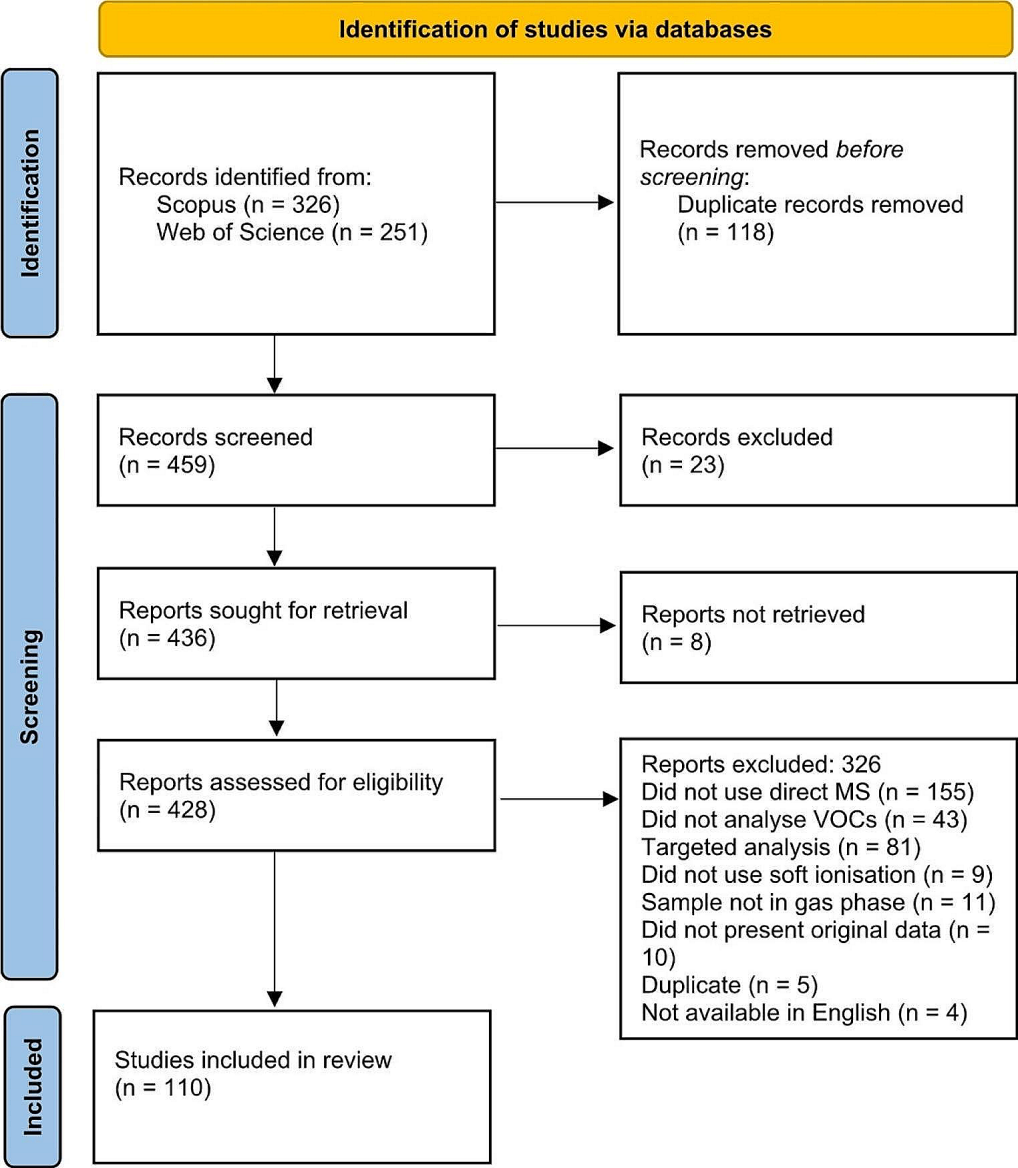
Flow diagram of the literature selection process. Database searches and record screening were performed automatically. Retrieval was performed automatically, then a manual process was performed if the paper had not been initially retrieved. Reports not retrieved were either not available online, or in a journal that Loughborough University does not provide access to. The final assessment for eligibility was performed manually. Some of the papers excluded may have not met multiple aspects of the criteria; the reason listed is simply the first reason for exclusion identified

Figure 1 depicts the literature search process and the number of papers included/excluded at each stage. A total of 110 studies were included in the review.

### Sample types

**Fig. 2:**
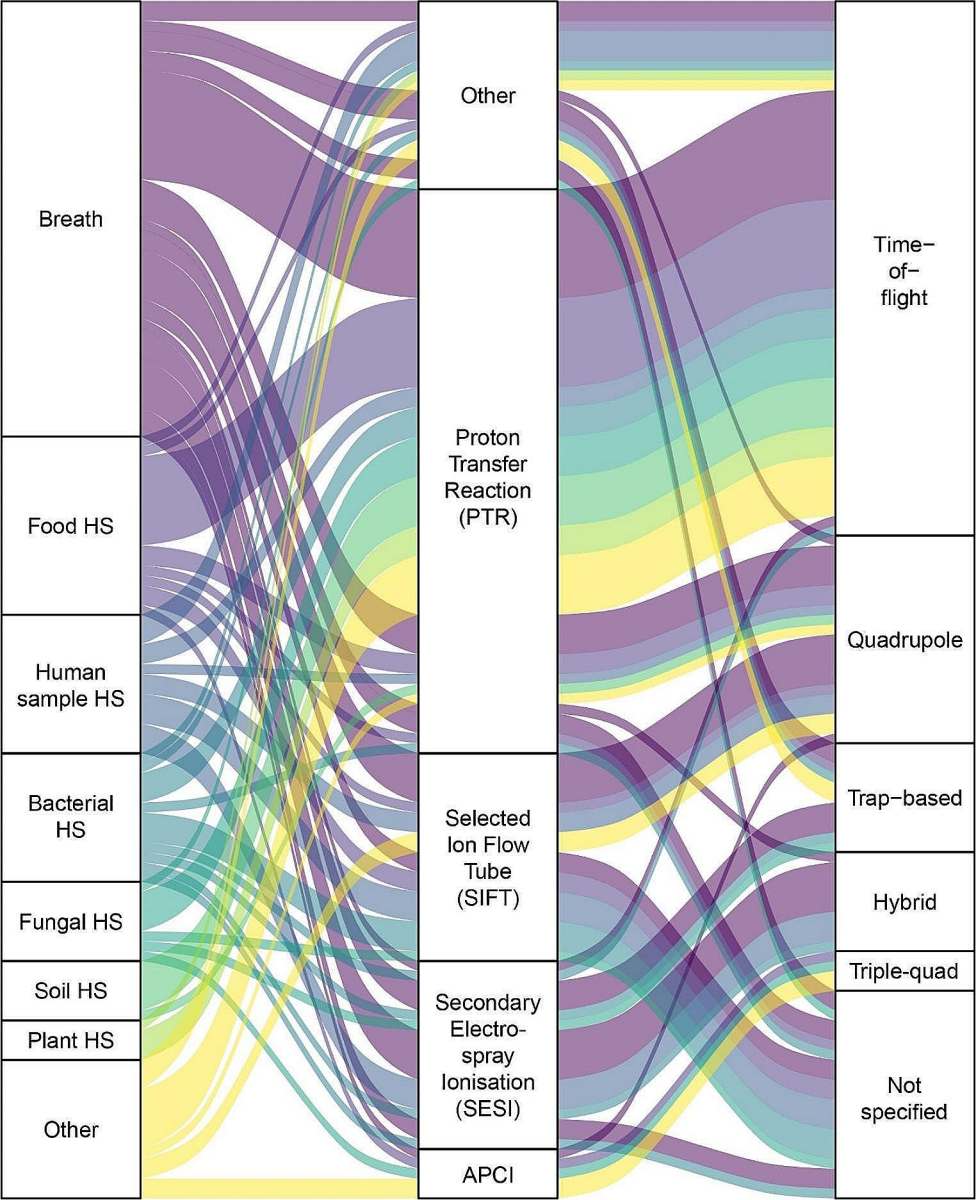
A Sankey network of the sample types and analytic methods used in the studies in this review. Studies which examined multiple sample types are represented separately for each sample type. HS = Headspace. APCI = Atmospheric Pressure Chemical Ionisation. The specific details of methods classed as ‘Other’ are available in the supplementary information

Figure 2 shows the distribution of sample types and which mass spectrometry methods. Breath was the most common sample type, with 40 studies (36.4%) which examined human breath and five (4.5%) murine breath. Nineteen studies (42% of all breath studies) measured human breath directly and continuously, typically by participants breathing through a mask which directed their exhalations towards the instrument through a transfer line. This results in spectra with peaks and troughs, corresponding to periods of exhalation and inhalation, respectively. Four studies analysed single breaths directly, while others collected single or multiple exhalations, which could be stored and analysed later, in bags (*n* = 12), vials (*n* = 2), Bio-VOC tubes (*n* = 2), or SPME fibres (*n* = 1).

Many studies analysed sample headspace, often of food (*n* = 18), bacteria (*n* = 13), or fungus (*n* = 8). Samples which release VOCs into the air can be analysed non-destructively by measuring the atmosphere above the sample, known as the headspace (Li et al., 2016). This allows the sample to be analysed multiple times, or continuously monitored, taking advantage of the ability of direct MS to analyse samples very quickly.

### Mass spectrometry methods

Table 1 shows how many papers met the minimum reporting guidelines for mass spectrometry analysis, proposed by the Chemical Analysis Working Group (Sumner et al., 2007). If an aspect was not included in the methods but could be reasonably inferred from elsewhere in the paper (for example, a figure showing spectra from m/z 0-300 was considered to be providing the mass range), it was deemed to have been reported. As different instruments have different adjustable parameters, it is difficult to determine whether all necessary parameters were reported, so this was considered to have been met if multiple pressure, voltage, temperature, and/or gas flow values were reported. The guidelines include the ionisation method and the acquisition mode (whether full scan or selected ion monitoring was used). As this review is only examining soft ionisation methods and untargeted data, by default, these have been reported by every paper included.

**Table 1:** The number of papers which reported each experimental parameter as suggested by the minimum reporting standards by the Chemical Analysis Working Group (Sumner et al., 2007)

Specified experimental parameter	Number of reporting papers (out of 110)	Percentage (%)
Sample introduction method	65	59.1
Ionisation method*	110	100.0
Ionisation polarity	82	74.5
Instrument parameters e.g., vacuum pressure, capillary charge	73	66.4
Mass analyser type	92	83.6
Acquisition mode*	110	100.0
Scan rate	58	52.7
Scan range	95	86.4
Calibration compounds	34	30.9
Mass resolution	32	29.1
**Total number of papers meeting the minimum reporting standards for the data collection methods**:	3	2.7

* Experimental parameters which were required for inclusion/exclusion criteria of review

Only three papers included all ten aspects of the minimum reporting standards by Sumner et al. (2007), with 17 (15.5%) reporting nine and 43 (39.1%) reporting eight. These standards were written to facilitate experimental replication and enable the re-analysis and comparison of data by others. This demonstrates that there is an issue with replicability in this area. While the guidelines are 16 years old at the time of writing and many methodological developments have been made in years since, they still cover the basic requirements necessary for replication in this area. It may be that researchers are simply unaware of the guidelines, particularly if they are not primarily working within metabolomics. Journals and reviewers could encourage the use of the minimum reporting standards by including them in submission guidelines and ensure that all the necessary details are included during the peer review process.

PTR was the most common ionisation method employed (*n* = 55, 50.0%), with SIFT (*n* = 18, 16.4%) and secondary electrospray ionisation SESI (*n* = 17, 15.5%) also used by a high proportion of papers. All other ionisation methods were used in three or fewer papers. Time-of-flight was the most common type of mass analyser used (*n* = 54, 49.1%), and 20 studies (18.2%) utilized a Quadrupole instrument. Seventeen papers (15.5%) did not specify the type of mass analyser. The type of mass analyser will affect the mass resolution, quantitative ability, and the sensitivity of the measurement (Li et al., 2021); therefore, to accurately replicate a study, this information is vital.

Twenty-eight studies (25.5%) did not specify whether the instrument was operated in positive or negative mode. Of the remaining 82, 70 used only positive mode, one only negative, and 11 used both positive and negative mode. The polarity will change the resultant spectra (whether a particular analyte is measured at mass + 1 or -1) and can affect the ionisation efficiency, which will in turn effect the sensitivity and detection limit (Liigand et al., 2017). Therefore, it is important that this information is included so studies can be accurately replicated.

The mass range was typically low with the lower m/z limit ranging between 0 and 100 (median = 20) and the upper m/z limit from 100 to 2010 (median = 250), with fifteen papers (13.6%) not specifying a m/z range. VOCs typically have a low molecular weight of around 50–200 m/z (Rowan, 2011); therefore, these ranges are expected for untargeted VOC analysis.

### Data processing tools

Forty-five different data processing tools were used, with 65 studies (59.1%) using multiple types of software/programming languages. Nineteen papers (17.3%) did not mention any data processing tool. Computational tools which were used three or more times are listed in Table 2. All other tools used are available in the supplementary information.

**Table 2:** Computational tools for direct mass spectrometry data acquisition and analysis

Tool	Type	Number of papers	Percentage
R	Programming language	39	35.5
Matlab	Programming language	32	29.1
SPSS	Statistical analysis software	17	15.5
ToF-DAQ	Data acquisition software	9	8.2
Excel	Spreadsheet software	8	7.3
MSConvert	File converting software	7	6.4
Xcalibur	Data acquisition software	5	4.5
Sigmaplot	Data visualisation software	5	4.5
PTR-MS Viewer	Data acquisition software	5	4.5
PeakView	Data acquisition software	5	4.5
SIMCA	Multivariate statistical analysis software	4	3.6
Metaboanalyst	Web server for metabolomic data analysis	4	3.6
Labsyft	Data acquisition software	4	3.6
gplots	Package written in R programming language	4	3.6
ggplot2	Package written in R programming language	4	3.6
FactoMineR	Package written in R programming language	4	3.6
SAS	Programming language	3	2.7
PTR-TOF Data Analyzer	Statistical analysis software for PTR-TOF	3	2.7
Minitab	Statistical analysis software	3	2.7
ade4	Package written in R programming language	3	2.7

Many of the programs named were data acquisition tools such as ToF-DAQ (*n* = 9, 8.2%) and PTR-MS Viewer (*n* = 5, 4.5%), which are typically used as an interface between the instrument and computer, although they also often contain data visualization tools and some aspects of data processing. For example, PTR-MS Viewer can aid compound identification and create simple statistics such as average and maximum/minimum (Ionicon, 2023). PTR-MS Viewer is provided by Ionicon with the purchase of an instrument, so the choice to use a particular software may be influenced by the availability of manufacturer provided software.

The R programming language was the most commonly used data processing tool (*n* = 39, 35.5%), with MATLAB also used often (*n* = 32, 29.1%). Programming languages provide a wide range of options for data processing as custom code can be created to suit the needs of the researcher. However, this can make replicating analysis steps difficult as some processing steps can be performed in many different ways, compared to a piece of software with limited options, such as SPSS (*n* = 17, 15.5%) or SIMCA (*n* = 4, 3.6%).

To ensure future researchers can copy the method accurately, studies should list the packages used and, where necessary, the specific functions. Of the 66 papers (60.0%) which used at least one programming language, 34 (51.55%) listed at least one package, with a total of 66 different packages named, across a wide range of uses (e.g., plotting, scaling, and multivariate analyses). The most times any package was mentioned was four (FactoMineR, ggplot2, and gplots), with 54 (81.8% of all packages) only mentioned once. Only eight of the packages (12.1%) were specifically designed for metabolomics data (ChemometricsWithR, MALDI-QUANT, MetaboAnalystR, MetStaT, mixOmics, Peaks, ptairMs, and ToF data plotter), none of which were used more than twice. This demonstrates a gap in the available analysis tools for a package specifically designed for direct MS, which can be used widely across the field.

Open-source packages are a useful tool in increasing replicability of research, as researchers can copy a workflow closely by using the same functions included in the package. However, currently, researchers are using a wide range of packages and processing methods, demonstrating the need for a workflow that can be easily replicated, designed specifically for direct MS.

The easiest way to improve replicability of studies using programming languages is to include the code as supporting information. If a programming language has been used (such as R or MATLAB), the code scripts can be saved; therefore, researchers can copy the method exactly simply by importing their own data, then running the code. While 66 papers reported using a programming language for at least part of their analysis, only two had the majority required to perform the analysis available online, a further two had a small section of their processing available, and two more indicated that the code would be available on request. Therefore, 60 papers (90.9%) which used code made no indication as to the availability of the code scripts. While more code may be available if requests to the authors were made, this requires the authors to be contactable, which may not be possible if they no longer work within academia. To ensure longevity, the code scripts should be included in the supporting information or accessible via a code repository, with a link provided in the paper.

Similarly, raw data should be made available wherever possible. Only two papers had raw MS data readily available. Thirteen others indicated that the data would be available on request, and one other had some of their data available. Therefore, 94 papers (85.5%) did not indicate whether the raw data would be available. While other papers had processed data available, this is not adequate to allow for the complete re-analysis of the data. Journals and grant applications are increasingly requiring data management and availability plans, which should help facilitate the improved availability of datasets.

### Data pre-processing and pre-treatment

Table 2 lists potential data pre-processing and pre-treatment steps for untargeted direct MS and gives a description of how they were defined for this study, expanding on the terms listed by Goodacre et al. (2007) and only including those relevant to untargeted direct soft ionisation MS. Commonly used methods and the number of papers which specified a method for each step has been reported in Table 2. Without attempting to perform the method, it is difficult to determine equivocally whether enough details of the algorithm and all the required meta parameters have been provided; however, in most cases it appeared that not enough information had been provided, particularly when the specific software, packages, or functions were not provided, as described above.

For every step listed, the majority of papers did not describe whether or not it was performed. Only one paper specified that a step was not performed; Neyrinck et al. (2022) specified that the data was not normalised. Some studies mentioned a data processing step but did not specify the method. This was a particular issue for centring (*n* = 4) and scaling (*n* = 3), although it is very likely that the authors were referring to mean centring and autoscaling, which are the most common methods and the only methods recorded in this review. A total of 68 different data processing steps were extracted from the studies, most of which can be summarised by the terms listed in Table 3.

**Table 3:** Potential data pre-processing and pre-treatment steps for untargeted direct mass spectrometry and commonly used methods

Step	Description	Commonly used methods*	Papers which specified a method† (count (%))	Papers which did not mention the step† (count (%))
Data selection	Selecting only the parts of a run which contain relevant data, often by detecting a change in the intensity of a particular ion, or total ion count.	Breath tracker (*n* = 12)	16 (14.5%)	94 (85.5%)
Baseline correction	Correcting for instrumental drifts.	Polynomial fit baseline subtracted (*n* = 6)	8 (7.3%)	100 (90.9%)
Deconvolution	Resolving overlapping peaks.	Modified Gaussian functions (*n* = 8)	8 (7.3%)	102 (92.7%)
Peak picking/alignment	Peaks can be defined and assigned exact m/z values, with only values which appear above a baseline selected for further analysis, or data can be binned into windows.	Centroided (*n* = 8) · Binned into windows (*n* = 4)	19 17.3%)	91 (82.7%)
Value calculation	A typical run includes multiple scans. These scans are then usually summarised by a single value.	Average over multiple scans (*n* = 32) · Integration of area under the curve (*n* = 3)	37 (33.6%)	73 (66.4%)
Background subtraction	Removing the intensity measurements that are due to the background profile, not the sample.	Subtraction of blank sample (*n* = 29)	30 (27.3%)	80 (72.7%)
Noise reduction	Data may contain random instrumental noise which can be corrected for.	Replicate averaging (*n* = 20) · Resampling (*n* = 7) · Smoothing (*n* = 6) · Wavelet denoising (*n* = 3)	30 (27.3%)	79 (71.8%)
Methods-based normalisation	Correcting for systematic variation by scaling each sample to an additional measurement specific to that sample or batch which is expected to change proportionally to the ion intensity count.	Primary ion (*n* = 19) · Sample quantity (*n* = 7) · Instrument parameter (*n* = 6)	31 (28.2%)	78 (70.9%)
Data-based normalisation	Presenting data from each sample as a ratio, to compare proportional differences and reduce the impact of systematic variation.	Control condition (*n* = 9) · Maximum value (*n* = 4) · Total ion count (*n* = 3)	20 (18.2%)	87 (79.1%)
Transformation	Change the distribution of the data, typically to remove heteroscedastic noise.	Log transform (*n* = 22)	23 (20.9%)	87 (79.1%)
Centring	Performed on each column, makes the average value the same across columns, to reduce the influence of variables with high abundance on multivariate modelling	Mean centred (*n* = 12)	12 (10.9%)	94 (85.5%)
Scaling	Performed on each column to make the variance across columns similar, to reduce the influence of variables with high fold changes on multivariate modelling.	Autoscaling (*n* = 9)‡	9 (8.2%)	98 (89.1%)
Missing value replacement	Replacing missing values possibly due to detector dead time or low concentrations to improve statistical performance.	Poisson correction (*n* = 10)	14 (12.7%)	96 (87.3%)
Outliers	Data points which deviate from the distribution of the majority of the data and can have an undue effect on data analysis.	No method was used more than once.	2 (1.8%)	108 (98.2%)

* Methods used by three or more papers are listed. Details of other methods are available in the supplementary information

† Studies which specified that a step was not performed were not included in this count. Percentage is out of the total number of papers (110). Papers which did specify a method, did not necessarily provide enough information to replicate the method

‡ Autoscaling was assumed to refer only to the scaling method, although it is commonly used to refer to mean centring with unit (standard deviation) scaling (Goodacre et al., 2007). Two papers performed autoscaling but did not specify whether they also mean centred

Some techniques could be considered to cover multiple categories of data processing steps; for example, wavelet denoising could be deemed a baseline correction method as well as noise reduction and averaging over multiple scans could be considered a noise reduction and a value calculation technique. However, it would be possible for a study to perform a separate step for each term listed.

The order they are listed is not necessarily the order in which they were performed. Some steps by nature must be performed before others; for example, there must be a list of peaks or bins before they can be scaled. In some cases, the order in which they are performed may affect the outcome, such as background subtraction and missing value replacement, as background subtraction can sometimes result in missing values; therefore, if it is performed after missing value replacement, those new missing values will not be replaced. For some steps, the order in which they are performed will not affect the final dataset, such as centring and scaling. It was not possible to confirm, but generally, it appeared that studies listed the data processing steps in the order in which they were performed.

Direct MS allows for continuous breath sampling; however, the desired section of exhalation must be selected from a continuous breath trace which will include inspiration, dead-space and mixed exhalation phases. Of the 17 studies which measured breath continuously, 12 (70.6%) reported using a custom ‘Breath tracker’ algorithm to select the data corresponding to exhalations. Of the 12, five used Acetone as the tracker ion, a known component of breath (O’Hara et al., 2008), three studies did not specify which ion was used by the algorithm, while one each reported using the total ion count, the sum of 4-hydroxy-2,6-nonadienal (m/z 155.1067) and 4-hydroxy-2-decenal (m/z 171.1381), and CO_2_. Additionally, one study used a separate CO_2_ monitor to determine when the participant was breathing out by the increase in CO_2_ readings. Six papers cited Trefz et al. (2013) or their subsequent papers (Sukul et al., 2014, 2015) regarding their use of the MATLAB-based breath tracker; however, the code for this breath tracker does not appear to be publicly available.

The majority of studies performed multiple scans per measurement, then summarised these scans into a single spectrum. For GC-MS, the measurement along the time axis provides important information to identify and separate different compounds. However, for direct MS, the measurement in time is only relevant if continuous monitoring is being performed; even then, researchers may still choose to summarise multiple measurements to reduce the effect of instrumental noise. Only 37 studies (33.6%) described how these scans were summarised into a single measurement and one paper specified that only one mass scan was performed per sample. For most data processing steps, it could be assumed that if a study did not mention a step, it probably did not perform that step, although it cannot be confirmed. However, a value must have been calculated somehow, and this information is missing from 72 (65.5%) of the papers in this review.

Eight papers described ‘centroiding’ peaks; however, a centroid typically refers to a measure of central tendency across multiple dimensions, with mean or median generally used for a set of real numbers (Deakin et al., 2002). For chromatography-coupled MS, the joint measurements for retention time and mass-to-charge ratio can be used to calculate the centroid. To describe the central tendency of a set of mass-to-charge ratios as a centroid is not necessarily wrong, it is not the language typically used in this situation and lacks specificity. This shows that many of the methods are those used more generally for MS and are not adapted specifically for direct methods.

Only four papers described binning the data into *m/z* windows; however, 41 reported *m/z* values to whole units, suggesting that more studies had used binning without describing it specifically. Binning can be useful for low resolution instruments, where more accuracy does permit greater precision. Without the additional information provided by a separation method, it is difficult to determine which *m/z* values should be considered the same ion. Binning to unit mass can increase the chances that the same ions are grouped together and reduce processing time (Finch et al., 2022).

Replicate averaging was the most common method of noise reduction used (*n* = 8). This is a commonly used method within metabolomics(Broadhurst & Kell, 2006) and can be used to reduce the effect of random noise and, depending on the research design, instrumental drift. Resampling can similarly be used to sample across the noise within a sample run in cases where the measurement may be assumed constant across the run. Note that this is a method available in direct sampling that is not available when there is chromatographic separation, which introduces a separation-related time dependency even when the sample is constant. Sampling within runs was used in 7 cases. However, it appears most of the papers using resampling used those methods as part of a smoothing and alignment process commonly applied to chromatographic methods (Prince & Marcotte, 2006). This again indicates that many of the data processing methods are simply copied from chromatography-coupled MS methods, rather than specifically designed for direct MS.

In most cases, only one method for each data processing step was performed; however, some studies used multiple methods within the same category, such as correcting by primary ion count and an instrument parameter (*n* = 3). Of the 19 papers which normalised by the primary ion (and the primary ion water cluster in seven papers), 14 used PTR-MS, four used SIFT-MS, and one used selective reagent ion-MS, which is effectively a combination of the PTR and SIFT methods (Jordan et al., 2009). The primary ions (those used to ionise the analytes of interest) for these methods can be measured by the mass analyser and this has a direct relationship to the measurement of product ions (Tani et al., 2004). This is not possible for every type of ionisation, as it requires primary ions which can be measured by the mass analyser.

Seven papers cited the Cappellin et al. (2011) workflow to describe their data processing methods and another cited their subsequent paper (Cappellin et al., 2012) which uses similar methods; polynomial fit baseline correction, modified Gaussian functions to define peaks, and Poisson correction for instrument dead times. All of these papers used PTR-TOF-MS, suggesting that the method may be specific to that analysis method. Additionally, seven of the eight papers had at least one author in common with the cited paper, suggesting that there hasn’t been much uptake of the method outside of the research group. Forty-two papers used PTR-TOF-MS; therefore, only 19% of PTR-TOF-MS used this workflow, or parts of it.

Using the same methodological workflow allows for results to be directly comparable, making it easier to review the literature in a specific area. Wherever possible, standardised workflows should be used to ensure this. However, the development of data processing workflows has not matched the development of new instrumentation. Additionally, the workflows must be accessible to new users. Cappellin et al. (2011) does not provide detailed instructions on how to replicate the workflow, nor any software which would be easy for a non-expert to implement, although they state that the MATLAB code is available on request.

Apart from the use of the workflow by Cappellin et al. (2011) and the use of primary ion normalisation, the data collection methods do not appear to have a large influence on which data processing methods were chosen. They were no clear patterns of data processing methods depending on the sample type, ionisation method, or type of mass analyser. Similarly, there were no other obvious groups of data processing methods that were consistently used together.

The methods chosen for transformation, centring and scaling will depend on whether they are relevant to the subsequent data analysis. Transformations are typically used to make the data conform to a normal distribution and reduce the impact of heteroscedastic noise. This may not be necessary for non-parametric methods. A log transformation can also be used to improve the visualisation of the data. Centring and scaling will not affect univariate data analysis and only studies which performed multivariate data analysis centred or scaled the data. Of the 72 studies which performed multivariate data analysis, 18 (25%) centred and/or scaled the data.

Generally, not enough detail has been provided for the data processing and statistical methods to be replicated easily. Many terms used are quite general, such as ‘wavelet denoising’ or ‘batch correction’, and without the specific method applied, cannot be replicated. Some papers described part of a step but not all, such as ‘peaks were centroided’. This generally the last step of peak picking, but it does not describe how the peaks were initially defined. Additionally, many methods have user-defined parameters which may affect the results, such as the order of a polynomial or the number of iterations in a machine learning technique. How these are defined is important information to ensure accurate replication so must be included in the methods, as recommended by Goodacre et al. (2007).

### Data analysis methods

Four papers estimated the required sample size to appropriately power the statistical tests prior to conducting the study, and one paper calculated power post-hoc. Sixty-eight papers (61.8%) performed either univariate significance tests or bivariate correlations but of those only 25 (36.8%) specified a multiple comparison p-value correction. For untargeted analysis, there are usually hundreds of variables, meaning there is a high likelihood of type I error (false-positive) because some of the variables will be different by chance. Multiple comparison corrections reduce the possibility of making a type I error. Thirteen papers used a false discovery rate (FDR) correction, and 12 studies used a family-wise error rate (FWER) correction.

Seventy-one (64.5%) studies performed at least one type of multivariate analyses. Thirty-eight studies (34.5%) performed a factor analysis, with Principal Components Analysis (PCA; *n* = 33) the most common method. Seventeen papers (15.5%) performed a cluster analysis or similarity network and eight studies (7.3%) created heatmap visualisations.

Forty-two studies (38.2%) performed at least one classification modelling analysis of which twenty-six used machine learning techniques such as Partial Least Squares-Discriminant Analysis (PLS-DA; *n* = 15), Random Forests (*n* = 8), and Support Vector Machines (SVM; *n* = 7). Six studies used multiple methods of classification modelling. Classification modelling is generally a ‘supervised’ method, with the algorithm designed to maximise the differences between groups. However, this increases the risk of overfitting, as spurious differences can be used to accurately separate the groups, but they would not necessarily be present in the wider population (Broadhurst & Kell, 2006). This makes validating models on a separate dataset very important, and generally is considered best practice (Brereton, 2006; Broadhurst & Kell, 2006; Goodacre et al., 2007).

Out of the forty-two which performed classification modelling, only 11 studies (26.2%), used a separate validation set to ensure that their models were accurate outside of the data used to create the model. A further nine used cross-validation (*n* = 6), bootstrapping (*n* = 2), or permutation testing (*n* = 1) to validate their models; however, the validation set is not truly separate in these methods. 52.4% of the studies which implemented classification modelling did not perform any validation on their models.

Including every variable in a multivariate model can lead to overfitting, so reducing the number of variables included is important to ensure models are applicable to the wider population. Prior to multivariate analysis, 54 (out of 71, 76.1%) preformed at least one variable selection step. Forty-six studies selected variables using a univariate method by examining each variable individually against a threshold, such as using univariate significance tests (*n* = 28), or the ratio between inhalations and exhalations (*n* = 11). Multivariate methods of variable selection, where the combined effect of variables was examined were used by 36 studies. Some used correlations to remove closely correlated variables (*n* = 7). Stepwise variable selection (*n* = 5) and Lasso penalisation were also used (*n* = 4). Some multivariate modelling methods include specific output parameters which can be used to select variables such as the PLS-DA VIP score (*n* = 9). Eighteen studies used both a univariate and a multivariate variable selection method.

Thirteen papers (11.8%) reported a measure of reliability (or repeatability) for their methods, with 11 papers reporting the coefficient of variation and two using Lin’s concordance coefficient. This is particularly important to report for new methods to ensure differences detected are true differences, not simply random variation.

### Strengths and limitations of this study

To the best of our knowledge, this is the first systematic review of data processing and data analysis methods for direct MS. This review has identified a lack of standardisation in the methods applied and the detail of reporting. The use of previous workflows appears to be limited, with very few papers citing previous work. The methods which are used most often could be used to form the basis of future workflows.

This review examined 110 studies, which is likely only a small subset of the research in this area since 2015. There may be many other papers which performed untargeted direct soft ionisation MS but which did not include the search terms in the title, abstract or keywords, potentially focussing on the application rather than the methods. It is possible that because of this, the studies included here may not be a true representation of the wider research in this area.

### Recommendations and implications for future research

Standardised methodology and reporting are necessary to ensure replicable research and wider use outside of research settings. There are many potential applications for direct MS in clinical and industrial settings, but these cannot be realised without robust methodology and replicable results. Currently, a wide range of data processing and analysis methods are being used, with no clear consensus within the literature. Additionally, many of the methods were developed specifically for chromatography-coupled MS, and do not appear to function the same for direct methods.

Further research should be undertaken to develop easy-to-follow data processing workflows specifically for direct MS. Initially, this should include testing various data processing methods to determine which result in the most reliable datasets, by reducing the impact of systematic and random variation. For example, the relationship between an instrument parameter and the ion count must be established to calculate an appropriate normalisation factor. Recommendations cannot be made if the methodology has not been proven to improve the quality of the data. This is beyond the scope of this review. An optimised workflow can then be developed, and guides, packages and applications can be created to allow ease-of-use for non-experts. It may be fruitful to develop new collaborations with statisticians and data scientists to move this work forward.

Researchers may be unaware of best practice for reporting, particularly if they are using direct MS to facilitate research in a different area. The wide range of available methodologies means a non-expert is unlikely to be aware of every method, and the required detail to ensure reproducibility. Tools such as a clear reporting framework with links to explanatory material should be developed to facilitate standardised reporting; journals could include links to these in their submission guidelines to ensure broad uptake.

In the short term, researchers should aim to include more details of their processing and analysis methods, including when a step has not been performed, to ensure replication is possible. Where feasible, datasets and code should be available as online supplementary information. Additionally, the packages and functions used within the code should be listed in the methods. Journals and reviewers should take steps to ensure the guidelines by Sumner et al. (2007) and Goodacre et al. (2007) are followed, until updated guidelines and tools are available.

## Conclusion

This systematic review demonstrates the wide range of data processing and analysis methods currently used for untargeted direct soft ionisation MS, and the improvements that need to be made in order to ensure that studies can be replicated, and results compared. There is a lack of standardised workflows and those that do exist do not have a broad uptake, and are not generally adapted specifically to direct MS. The methods highlighted in this review can be used as a starting point in developing new workflows, suitable for a range of applications within untargeted direct soft ionisation MS metabolomics research.

Many papers do not meet the recommended minimum reporting standards set by Sumner et al. (2007) and Goodacre et al. (2007). The use of a standardised reporting tool, recommended by journals, will ensure these standards are met and that research in this area is reproducible. This will allow for the wide-ranging potential applications of direct MS to be realised.

## Data Availability

The extracted data is available in the supplementary information at 10.17028/rd.lboro.25358464.
